# Ex vivo expansion and characterization of human corneal endothelium for transplantation: a review

**DOI:** 10.1186/s13287-021-02611-3

**Published:** 2021-10-30

**Authors:** Ingrida Smeringaiova, Tor Paaske Utheim, Katerina Jirsova

**Affiliations:** 1 Laboratory of the Biology and Pathology of the Eye, Institute of Biology and Medical Genetics, First Faculty of Medicine, Charles University and General University Hospital in Prague, Albertov 4, 128 00 Prague, Czech Republic; 2 Department of Medical Biochemistry, Oslo University Hospital, Oslo, Norway; 3 Department of Plastic and Reconstructive Surgery, Oslo University Hospital, Oslo, Norway

**Keywords:** Corneal endothelium, Cell culture, Tissue engineering, Storage, Transplantation, Endothelial phenotypic markers

## Abstract

The corneal endothelium plays a key role in maintaining corneal transparency. Its dysfunction is currently treated with penetrating or lamellar keratoplasty. Advanced cell therapy methods seek to address the persistent global deficiency of donor corneas by enabling the renewal of the endothelial monolayer with tissue-engineered grafts. This review provides an overview of recently published literature on the preparation of endothelial grafts for transplantation derived from cadaveric corneas that have developed over the last decade (2010–2021). Factors such as the most suitable donor parameters, culture substrates and media, endothelial graft storage conditions, and transplantation methods are discussed. Despite efforts to utilize alternative cellular sources, such as induced pluripotent cells, cadaveric corneas appear to be the best source of cells for graft preparation to date. However, native endothelial cells have a limited natural proliferative capacity, and they often undergo rapid phenotype changes in ex vivo culture. This is the main reason why no culture protocol for a clinical-grade endothelial graft prepared from cadaveric corneas has been standardized so far. Currently, the most established ex vivo culture protocol involves the peel-and-digest method of cell isolation and cell culture by the dual media method, including the repeated alternation of high and low mitogenic conditions. Culture media are enriched by additional substances, such as signaling pathway (Rho-associated protein kinase, TGF-β, etc.) inhibitors, to stimulate proliferation and inhibit unwanted morphological changes, particularly the endothelial-to-mesenchymal transition. To date, this promising approach has led to the development of endothelial grafts for the first in-human clinical trial in Japan. In addition to the lack of a standard culture protocol, endothelial-specific markers are still missing to confirm the endothelial phenotype in a graft ready for clinical use. Because the corneal endothelium appears to comprise phenotypically heterogeneous populations of cells, the genomic and proteomic expression of recently proposed endothelial-specific markers, such as Cadherin-2, CD166, or SLC4A11, must be confirmed by additional studies. The preparation of endothelial grafts is still challenging today, but advances in tissue engineering and surgery over the past decade hold promise for the successful treatment of endothelial dysfunctions in more patients worldwide.

## Introduction

**Fig. 1:**
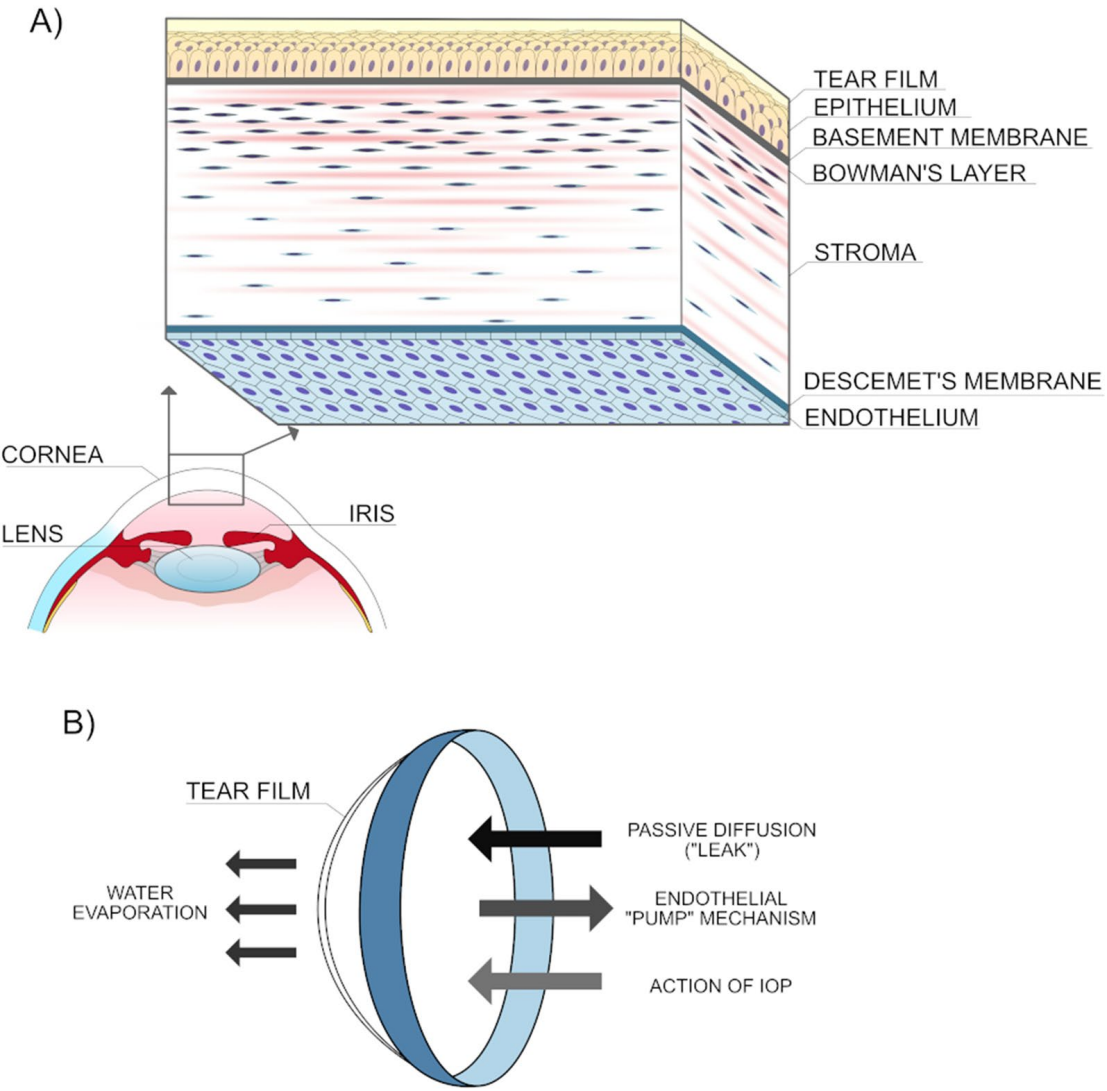
Anatomical layers (**A**) and fluid flow (**B**) in the human cornea. The corneal endothelium is the innermost corneal monolayer, formed mostly by hexagonal cells (**A**). The corneal endothelium controls corneal hydration by the so-called pump-leak mechanism (**B**). *IOP* intraocular pressure. Illustrations: Sara Tellefsen Nøland, IS

A transparent cornea is essential for vision because it mediates the entry of light into the eye. The human cornea is composed of six well-organized layers (Fig. 1A): the epithelium and its basement membrane, the Bowman layer, stroma, Descemet's membrane (DM), and the corneal endothelium (CE), each of which contributes to the correct function of the cornea and, thus, to good vision. The cornea contains three main types of cells: epithelial cells, stromal keratocytes, and corneal endothelial cells (CECs).

The CECs, which form a monolayer of polarized, mostly hexagonal cells that lie on the DM, influence the transparency of the entire cornea because its main function is to maintain adequate hydration (and thickness) of the corneal stroma (Fig. 1B). In the case of CE dysfunction, the inflow of fluid into the stroma predominates over its outflow, leading to excessive corneal hydration and disruption of the uniformly spaced stromal collagen fibrils, which changes the cornea’s optical properties. During human life, there is a gradual reduction in endothelial cell density (ECD) of approximately 0.6% per year, leading to a decrease in ECD from about 6000 cells/mm^2^ after birth to roughly 2300 cells/mm^2^ at age 85 years [1]. An ECD of more than 500 cells/mm^2^ is necessary for correct CE function [2, 3], and an ECD of 2000–2500 cells/mm^2^ is required for donor corneas intended for penetrating keratoplasty surgery [4].

**Fig. 2:**
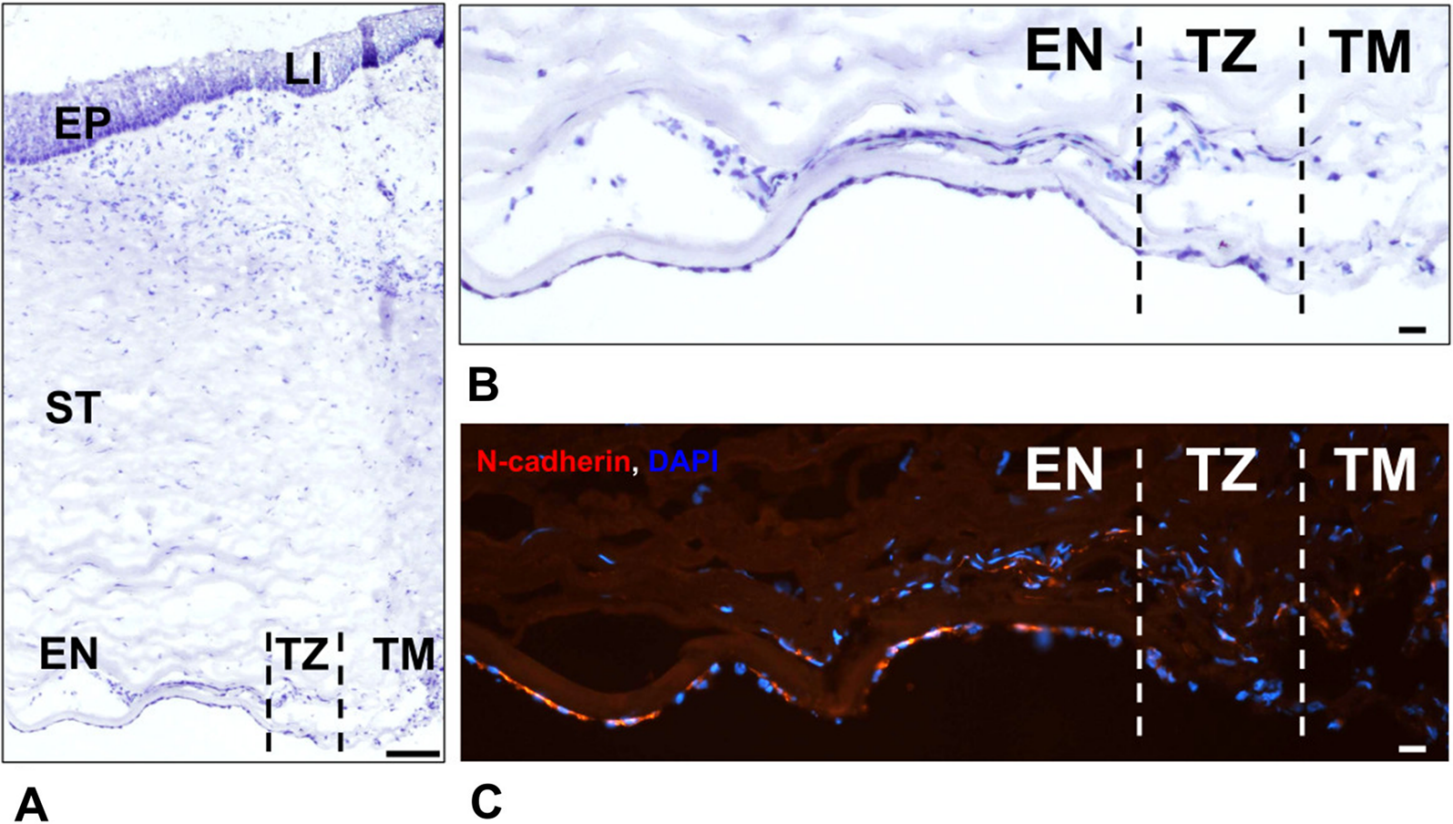
Peripheral endothelium and transition zone in normal human cornea. Light microscopic image of a healthy human cornea stained with hematoxylin and eosin (H&E) (**A**). Posterior cornea periphery in detail, H&E-stained (**B**). Detail of the posterior corneal periphery immunoassayed for the corneal endothelial marker N-cadherin (red); nuclei are counterstained with DAPI (blue) (**C**). *EP* corneal epithelium, *LI* limbal area, *ST* stroma, *EN* peripheral endothelium, *TZ* transition zone, *TM* trabecular meshwork. Scale bars: 100 μm (**A**), 20 μm (**B**, **C**). Source: Authors’ (IS, KJ) archive

Minor loss of CECs is repaired by cell migration and spreading of vital cells surrounding the denuded DM until the barrier and pump functions of the CE are restored [5]. The limited proliferation of stem/progenitor cells, which are thought to be located in the periphery of the posterior cornea [6–8], including the transition zone (TZ) and trabecular meshwork (TM) (Fig. 2), seems to contribute to CE barrier integrity as well. Adult human CECs, in both central and peripheral regions of the CE, retain their proliferative capacity [9], but under physiological conditions, CECs do not proliferate in vivo [5]. The proliferation of adult CECs has been observed in corneas with a wounded CE [6, 10] and in CECs cultured ex vivo [11]. Dysfunction or extensive loss of CECs, due to endothelial disease or trauma, is standardly treated by surgical replacement (i.e., by penetrating keratoplasty or, less invasive, lamellar keratoplasty). However, the global supply of donor corneas is low (only 1 cornea available for 70 patients), and approximately one-third of donor corneas is discarded due to worsened endothelial quality (such as low ECD) or the presence of infection [12]. Thus, the development of alternative or complementary methods of treatment CE dysfunctions is necessary. One option is a cell-based therapy, using the proliferative capacity of CECs and the presumed presence of stem/progenitor cells, which allow CECs to be propagated ex vivo by tissue-engineering (T-E) methods.

Endothelial tissue engineering is a challenge for several reasons. The CECs have a low natural proliferative capacity that must be stimulated in vitro with a mitogen-rich medium. During ex vivo expansion, CECs undergo senescence and phenotypic transformation to the mesenchymal cell type, so-called endothelial-to-mesenchymal transition (EndMT), which can be prevented by media supplements, such as inhibitors of selected signaling pathways. In addition, CECs are phenotypically highly heterogeneous because they originate in the neural crest and mesoderm [13, 14], and it is, therefore, difficult to find a specific marker for in vivo and ex vivo characterization of CECs to confirm their phenotype, particularly in T-E grafts.

So far, the cadaveric cornea appears to be the most suitable source of cells for ex vivo expansion due to a high similarity of genes expressed in cultured CECs, compared to CECs in vivo [15]. The most established cell isolation technique in current culture protocols consists of a “peel-and-digest” method, which includes peeling the CE on DM (CE + DM) from the donor cornea [16] and digesting it with collagenase or other nondestructive enzymes [11, 17, 18]. Then, cells are expanded via a “dual-media” approach of cultivation of CECs, which means the alteration of two phases (media) during culture (i.e., mitogen-rich (proliferation) and low-mitogenic (stabilization) media) [19]. This approach has led to the preparation of grafts successfully transplanted into animals, with promising results [20, 21].

However, due to a shortage of corneas from deceased donors, other cell sources for the preparation of CEC grafts are being investigated, such as immortalized endothelial cell lines, induced pluripotent stem cells, mesenchymal stem cells, embryonic or adult stem cells, as reviewed elsewhere [22]. Persistent problems with non-endothelial cells include their limited availability, ethical issues, the need for controlled reprogramming and differentiation into CECs, and the long-term preservation of endothelial phenotype and functionality without undesirable changes after transplantation (Tx), which is still challenging [23].

In addition to finding a suitable source of cells, tissue-engineering techniques consist of several crucial issues that affect the ultimate success of ex vivo endothelial culture: a sufficient number of vital donor CECs for in vitro culture, which is influenced by appropriate donor tissue storage and a gentle cell isolation technique; cell isolate purity (a requirement for the elimination of contaminating epithelial/stromal cells); culture conditions (appropriate cell substrate and optimized, xeno-free, culture media that support cell growth, morphology, and phenotype of CECs); a quality assessment of cultured CECs with specific molecular markers; storage and transport of T-E CE prior to grafting; and, finally, selection of the most suitable Tx technique. For future clinical application of T-E grafts, each of these steps must be well optimized.

Currently, there is no efficient and robust protocol for preparing T-E CE grafts for either lamellar keratoplasty or cell-injection therapy [22]. This review discusses several major aspects of the ex vivo preparation of CECs graft for transplantation purposes, focusing on human donor corneas as the most established source of cells in basic and clinical research. The goal of this paper is to summarize the direction and perspective of current culture protocols. We examine the literature, particularly in the field of basic research conducted in the last decade (2010–2021), on the ex vivo propagation of human CECs and on endothelial biology, including new endothelial-specific markers and possible signaling pathways involved in ex vivo CE culture.

This review shows that the introduction of a robust dual-media culture system for the ex vivo expansion of CECs, as well as intensive research into selected signaling pathway inhibitors, such as Rho-associated kinase inhibitors, has enabled the preparation of GMP-compliant CE grafts for first-in-human clinical trials with promising outcomes [24, 25]. According to the study, approximately 300 patients (eyes) can be treated using T-E grafts derived from one (younger) donor cadaveric cornea, using culture conditions developed by authors [26, 27]. Recent genome and proteome studies on CECs under in vivo and ex vivo conditions [18, 28] have brought new insights into CE biology, particularly into CE-specific markers that can potentially distinguish populations of healthy and functional CECs suitable for Tx purposes (cadherin-2, CD166, SLC4A11, etc.) [18, 26] from populations of phenotypically modified cultured CECs, unsuitable for clinical use (CD44, CD133, etc.) [26]. Further improvement of culture protocols, such as pre-cultivation of donor corneas in a low-mitogenic medium prior to cell propagation, can not only improve the quality of clinical-grade donor corneas (i.e., those with sufficient ECD and CE characteristics) intended for Tx [29] but also of research-grade corneas (i.e., those deemed unsuitable for Tx due to low ECD or CE parameters) for the subsequent ex vivo expansion of CECs [11]. Thus, such an approach can increase the global pool of donor corneas available for clinical use. An analysis of methods for preserving ex vivo propagated CECs has shown the possibility of storing these cells in suspension at very low temperatures without significant loss of vitality after thawing [30, 31], therefore allowing long-term storage and improving ready-to-use graft availability in the future.

## Corneal endothelium: biology and function

CECs are derived from the neural crest (neuroectoderm) and mesoderm [13]. CE development begins around the fifth week of gestation, under the influence of a wide range of transcription factors, such as Foxc1, Pitx2, and Pax6, but the precise mechanisms underlying these processes are not fully understood [32]. At birth, the CE forms a five-to-six-µm-thick monolayer of typical honeycomb-like cells, with large nuclei and numerous organelles, which persists throughout adulthood and can be monitored microscopically. A complex development of CE is reflected in the phenotypic heterogeneity of these cells; CECs share markers of neural (neuron-specific enolase, neural cell adhesion molecule [33]), epithelial (keratins (K) K8, K18 [34], K7, K19 [35]), or mesothelial (mesothelin, calbindin-2 [14]) cells.

CECs are highly organized, with apical sides bathed in the aqueous humor and basal sides connected to the DM by hemidesmosomes. The cells' membranes are interdigitated and contain focal tight junctions (zonula occludens), gap junctions, and adherent junctions, together with molecular pumps and transporters, such as Na^+^/K^+^-ATPase pumps, carbonic anhydrase, or sodium borate cotransporter 1 (SLC4A11), all of which are necessary for endothelial barrier and pump function [36]. The endothelial “pump function” is maintained primarily by the action of Na^+^/K^+^­ATPase pumps [36]. The tight junctions between adjacent CECs are not fully continuous and thus “leaky,” allowing fluid passage to the stroma from the aqueous humor [3, 36]. Because the CECs are highly metabolically active [37], the supply of nutrients from the aqueous humor is vital for their function. The CE function can be compromised by a number of factors that can result in corneal edema and vision loss. However, the CE has a natural self-renewing ability that involves the migration of CECs to cover the lesion (exposed DM), accompanied by increases in cell size variability (polymegathism) and cell shape variation (polymorphism). Thinning of the CE leads to an increase in the number and activity of endothelial pumps in the remaining CECs to maintain the CE function and, thus, a clear cornea [38].

The presence of endothelial stem cells in the cornea and the ability of CECs to proliferate are some of the most discussed topics in the literature in connection with the human CE. Human CECs do not proliferate in vivo because they are locked in the G1-phase of the cell cycle [5], but they can proliferate ex vivo [24, 39, 40]. Signal pathways involved in CEC proliferation are complex [41] and include those inhibiting proliferation (mainly transforming growth factor (TGF)-β/Smad) and those stimulating proliferation, migration, and wound healing, such as fibroblast growth factor (FGF)-2-PI3-kinase-Akt (also involved in the fibroblastic transformation of CECs), mitogen-activated protein kinase (MAPK), protein kinase C (PKC) activated via phospholipase Cγ, Wnt/Frizzled/β-catenin, Notch signaling (integrated with Wnt and TGF-β/Smad signaling), RhoA-Rho-associated protein kinase (ROCK) signaling (also participating in pathological fibroblastic changes of CECs), and also the TGF-β1/PKC pathway, as reviewed by Zhang et al. [41].

The presence of endothelial stem/progenitor cells in the cornea was predicted by the detection of general molecular markers such as nestin, alkaline phosphatase, telomerase, Sox-2, Oct-3/4, and Lgr5 [6, 7, 42] in the extreme periphery of human CE and TZ. Moreover, the ECD in the extreme periphery of the CE is approximately 10% higher than that in the central area [43], and those CECs have an altered morphology, such as a smaller spherical shape, compared to CECs in the central region of the CE [8]. However, the correct identification and isolation of stem/progenitor cell subpopulations from donor cornea and their ex vivo propagation and differentiation into functional CE remain difficult [44].

## Donor parameters and corneal endothelial quality

The quality of the endothelial in vitro culture, derived from cadaveric corneas, is significantly influenced by donor parameters such as health status, age, cause of death, time from death to the establishment of the primary cell culture, and type of tissue storage prior to culture [11, 39].

In general, CECs harvested from younger corneas are more easily expanded in vitro than those from older corneas [10]. Younger CECs show better adherence to culture substrate, higher proliferation rates in vitro, and can reach a higher number of passages without chromosomal aberrations or senescence [45, 46]. Despite worsened response to mitogenic stimulation in culture [5], expansion of CECs from older corneas is possible using novel T-E techniques, such as forced cell attachment after seeding [45]. Nevertheless, it has been observed that CECs of older donors have an altered expression of proteins important for cell metabolism, protein folding, and antioxidant protection compared to younger donors [47]. With regard to the isolation of CE + DM lamellae from cornea, older donor corneas appear to be a better source of tissue than younger ones because they allow easier removal of the lamella from the cornea due to a higher age-related DM thickness [48].

Older donor corneas contain more senescent CECs with truncated telomeres and damaged DNA than younger corneas [5]. This observation may be related to oxidative stress induced by high CEC metabolism rates [49] and ultraviolet (UV) light [50]. CECs that reside in the thickened corneal periphery are protected more effectively from UV radiation than central CECs [50]. Much of the UV light is absorbed by the corneal epithelium and tear film. CECs are also protected by antioxidants present in the aqueous humor and cellular antioxidants (peroxiredoxin 6, glutathione peroxidase 4) [51].

## Storage of donor tissue prior to use

The donor corneas for Tx can be stored either under hypothermic conditions (HT) at 2–8 °C or in organ culture (OC) at 30–37 °C, as reviewed by Jirsova et al. [29]. Donor corneas intended for Tx are more often stored under HT for ease of storage and immediate availability of tissue for Tx; the corneas stored in OC swell and must be de-swelled before use.

Prolonged HT (over 14 days) has a negative effect (e.g., induction of apoptosis and necrosis) on CE quality [52]; thus, donor corneas intended for Tx are stored for shorter periods [29]. On the other hand, the OC allows prolonged storage of up to 4–5 weeks [29] and contributes to better outcomes of in vitro cultures than HT [11, 53] because it supports cell migration and possibly also repair of CE [11, 29, 54]. The loss of CECs during HT tissue storage can be reduced by adding caspase inhibitors, which improve CEC tolerance to cold stress under HT [55] or by anti-apoptotic gene therapy [56]. Novel approaches include storing the donor corneas in bioreactors mimicking the internal environment of the eye [57] for maintenance of CE quality.

## In vitro expansion of cells from donor tissue

### Donor cell isolation

For cell-based therapies, most of the current culture protocols isolate CECs either from research-grade corneas or corneoscleral rims, remaining after Tx [20, 39]. The age of the donors varies, but mostly younger donors are used in ex vivo expansion studies due to the properties of the younger tissue.

The most commonly used method of harvesting CECs involves a two-step isolation procedure termed the “peel-and-digest” method [16], which includes manual peeling of the CE + DM from the cornea and dissociation of cells into cell clusters and single cells by enzymatic digestion. This method is more common and more effective than the explant method [17]. The most common enzymes used for cell isolation involve collagenase (A or Type I), TrypLE™ Express/Select or Accutase [11, 17, 18]. Digestion with trypsin, trypsin/EDTA, dispase, or EDTA only was shown to be inefficient [16, 17] or induced changes in endothelial phenotype [58]. For example, Li et al. [16] reported that a brief (10 min) treatment of CECs with trypsin/EDTA following initial digestion with collagenase can promote CEC proliferation, but, in other study, subsequent culture in FGF-2-containing medium induced EndMT transformation of CECs [58]. A recent comparative study showed that both collagenase and trypsin can lead to confluent and functional CEC cultures (derived from ˂ 40-year-old donors), but collagenase-isolated CECs formed a slightly stronger CE barrier than CECs isolated by trypsin [18].

During CEC isolation, other corneal cells, particularly stromal keratocytes, may be co-isolated and, thus, contaminate the CEC culture. This issue is addressed in several ways [22]; for example, CEC and keratocyte populations may be separated by flow cytometry cell sorting, based on the specific phenotypic markers, or the sedimentation field flow fractionation method, based on cell sorting according to the biophysical properties without the use of antibodies.

The isolation and subsequent ex vivo propagation of CECs are accompanied by non-physiological environmental stress placed on cells, which is reflected by rapid cell death. This stress may significantly reduce the number and quality of CECs available for culture, particularly when the cells are isolated from the tissue with low ECD. An effective approach that has been implemented in culture protocols over the past decade involves the addition of a step of pre-cultivation of dissociated CE + DM lamellae or cells after enzymatic isolation in a low-mitogenic stabilization medium for one to several days prior to cell seeding. This method improves the survival and morphology of CECs during subsequent in vitro expansion [11, 18, 59]. Ex vivo culture outputs are also increased by high initial seeding density (> 100 cells/mm^2^) [26, 60] or by combining CECs from paired donor corneas into a single batch to increase cell numbers for culture [45]. The lower adhesion of CECs, especially those isolated from older donor corneas, can be helped by loading the cells with a viscoelastic solution after seeding [45].

### Cell substrates

The substrate composition and topography have a crucial impact on the success of CEC culture because they stimulate the attachment, migration, proliferation, and overall function of the cultured CECs [17, 61]. Due to the poor adherence of CECs to uncoated culture dishes, various biological, biosynthetic, or synthetic substrates for CEC expansion have been tested up to now, as reviewed elsewhere [62–64] (Table 1). The ideal substrate should mimic the composition (collagens 4–6, 8, 18, fibronectin, laminin, thrombospondin) and topography of DM [64, 65], therefore helping to maintain the CEC phenotype and function and promoting CE regeneration after injury [66]. It was shown that a replacement of the DM alone can be sufficient to induce the regeneration of host CECs in vivo [67]. In recent studies, an FNC coating mix® [11, 40, 45] and collagen 1 and 4 [21, 62, 68] have been the most widely used substrates for CEC cultures.

**Table 1:** Some substrates used for in vitro expansion of corneal endothelial cells (CECs)

Substrate	Properties in relation to corneal endothelial cells	Ref
**Biological substrates**		
Decellularized human corneal stroma	Support formation of confluent CE monolayer, and the maintenance of the CE phenotype	[118]
Denuded Descemet's membrane (DM)	The denuded DM + CE construct rolls in the opposite direction (with CE inwards) than the CE + DM lamella (with CE outside) in solution, which complicates Tx. Tested on human primary CECs	[119]
Human amniotic membrane (HAM)	A non-immunogenic carrier, composed of collagen 4, which supports CEC proliferation and differentiation. The semi-transparent nature and variable quality of the tissue limit the use of HAM for the purpose of Tx. Moreover, in rabbit eyes edema was observed seven days after Tx of CE grown on HAM	[73]
Decellularized human lens capsule (DHLC)	Composed of collagens (1, 3, 4, 8), laminin and fibronectin. Facilitates CECs’ expansion and sustains the endothelial phenotype. DHLC + CE construct has a good adherence to posterior stroma after Tx. In solution rolls with CE inwards	[119, 120]
**Culture plate coatings**		
Collagen 1 or 4	Main proteins in the human cornea. Improve attachment and morphology of primary CECs; supposed to maintain the normal phenotype of CECs and prevent phenotypic change. Handling of soft collagen-based substrates may be improved by a cross-linking the collagen fibers	[62]
Laminin 5	Promotes adhesion, migration, and proliferation of human primary CECs (donor age: 55–76 years), and supports wound healing of injured CEC	[121]
Laminin-511, -521	Enhance adhesion, proliferation, and differentiation of human primary CECs (donor age: > 40 years)	[122]
Laminin E8 fragments	Support human CECs’ expansion with a similar efficacy to that obtained with laminins-511/-521. Recombinant laminin fragments can be produced more easily than full-length laminins	[53, 122]
FNC coating mix ® (fibronectin + collagen 1 + albumin)	Improves rapid attachment of primary human CECs and reduces cell loss due to rinsing of cells. Accelerating the CECs’ attachment more significantly than collagen I	[40, 45]
**(Bio)synthetic substrates**		
Collagen 4 + laminin-coated collagen 1	Supports the formation of confluent CE monolayers (human and bovine primary CECs) and the maintenance of the CE phenotype	[123]
Poly (lactic-co-glycolic acid)	Preserves morphology and high cell viability (on smaller fibers with smaller interstitial space); tested on HCEC-12 cell line	[61, 124]
Poly-ε-lysine (pεK) cross-linked with octanedioic acid	Supports adhesion, expansion and maintenance of the CE phenotype; tested on the HCEC-12 cell line and porcine CECs	[125]
Poly (D, L‐lactic acid) and cross-linkable gelatins	Supports proliferation and correct phenotype of cultured CECs; tested on primary human CECs and the B4G12 cell line	[126]
Poly (glycerol sebacate) with poly (ε-caprolactone)	Supports the formation of confluent CE monolayers and the maintenance of the CE phenotype; tested on HCEC-12 cell line and human conjunctival epithelial cells. This biodegradable scaffold is semi-transparent, non-immunogenic and highly elastic	[127]

Another rather innovative approach in the cultivation of CECs is the usage of a medium that temporarily prevents CEC attachment to the substrate, the so-called sphere-forming assay [69, 70]. This method aims to isolate and maintain precursor (stem/progenitor) cells under non-adherent conditions to form floating beads, which can then form functional CE monolayers under appropriate culture conditions (e.g., in a medium with serum) [71]. Human precursor cells, derived by sphere-forming assay, were able to restore CE function after Tx into the rabbit eye [72]. However, the sphere-forming assay for CE precursors remains challenging and, thus, has yet to become established.

### Culture media

Several culture media have been used for CEC expansion. These media include Dulbecco’s modified Eagle’s medium (DMEM) [46, 73], a mixture of DMEM with Ham’s F12 (F12) supplement [46], M199:F12 (1:1), also referred to as F99 medium [39, 46], endothelial growth medium 2 [17], endothelial serum-free medium (Endo-SFM) [74], and Opti-MEM I [40, 46, 62], which is reduced serum minimal essential medium, enriched for insulin, transferrin, hypoxanthine, thymidine, and trace elements. Currently, the most used media for CEC proliferation are F99 and Opti-MEM I [40, 46]. Another medium, the supplemented hormonal epithelial medium, composed of DMEM/F12 (1:1), fetal bovine serum (FBS), epidermal growth factor (EGF), dimethyl sulfoxide (DMSO), and cholera toxin [16], can also yield confluent CE monolayers, even at an initial low seeding density [16], but it is less efficient than Opti-MEM I or F99 [46].

**Fig. 3:**
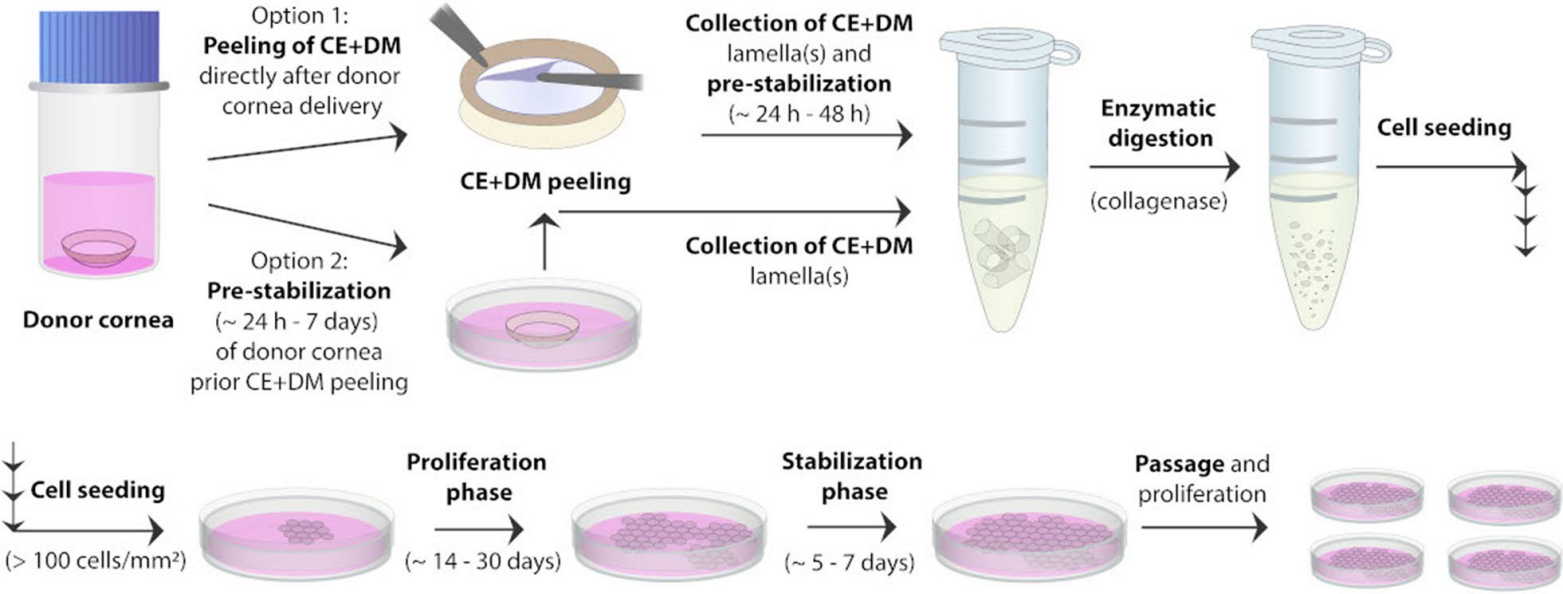
Scheme of currently used in vitro culture of human corneal endothelial cells. Corneal endothelium on the Descemet's membrane (CE + DM) is derived from cadaveric donor cornea using the peel-and-digest method. Manual peeling of CE + DM can be performed immediately after tissue delivery, followed by pre-stabilization of isolated lamella(s) at 37 °C (Option 1), or pre-stabilization of intact donor cornea can precede the CE + DM peeling (Option 2). After peeling, the collected lamellae are enzymatically digested (typically with collagenase A or Type I at 37 °C) and cell clusters disintegrated by second digestion with, for example, TrypLE™ Express/Select solution. Cells are then seeded onto a suitable cell substrate at concentrations of > 100 cells/mm^2^ and expanded using the dual-media culture approach (switching proliferation and stabilization media). At the end of the culture period (at approximately 80% confluence), the cells can be passaged and further expanded, again using the dual-media culture system. Illustrations: Sara Tellefsen Nøland, IS

The most widely used culture methods involve the “dual-media” approach, which includes alterations of two culture media and two different culture periods [19], as shown in Fig. 3. The first (proliferation) phase lasts for several weeks and includes a proliferation medium (PM) containing FBS (> 5%) and mitogens for CEC expansion (typically up to 80–90% confluence). The following “stabilization” phase is shorter (~ 2–7 d) and includes a low-mitogenic stabilization medium (SM) with antibiotics; this medium is used to stabilize the CEC phenotype before cell isolation from dissected CE + DM lamella or prior seeding of isolated cells onto a substrate for primary culture or passages. Prolonged culture of CEC in PM leads to a gradual loss of hexagonal morphology, whereas culturing CECs in SM leads to the maintenance of CEC morphology but insufficient cell growth [18]. From a clinical point of view, only CECs expanded up to the first passage are considered the most appropriate for Tx because, with higher passages, CECs undergo senescence and phenotypic changes in both low and high mitogenic conditions [18].

Culture (proliferation) media are standardly supplemented with components that support cell attachment to the substrate (e.g., ROCK inhibitors), proliferation (various growth factors), or inhibit oxidative aging (antioxidants) or EndMT (inhibitors of selected signaling pathways). Most of the current culture media formulations for CEC expansion contain xenogeneic components, such as animal serum, which makes such media incompatible with strict clinical requirements. This problem can be partially solved by the substitution of animal serum with human serum [75], modified platelet lysate [76], or other recombinant proteins and small-molecule compounds [77] that have recently been used for CE ex vivo expansion.

Another important component of culture media is growth factors (GFs). The effect of some GFs, such as insulin-like growth factor (IGF) [78] or nerve growth factor [79], on cultured CECs appears to be marginal, whereas the effects of EGF or FGF-2/bFGF are more significant. Both EGF and FGF-2 have dual effects of CECs; the EGF was shown to stimulate CECs to enter the cell cycle [10, 79] and promote healing of the CE layer [80] but, in conjunction with TGF-β, also stimulate EndMT [81], and FGF-2 either promotes CEC proliferation (via the PI3K and ERK1/2 → p27 pathways) and migration (by activating CDC42 with PI3K/p38) but also stimulates EndMT (via Rho GTPase and overexpression of the transcription factors SNAI1 and ZEB1) [10, 82]. SNAI1 transcription factor regulates both FGF2-dependent cell proliferation and EndMT, acts upstream of ZEB1—induces ZEB1 and CDK2 in parallel, leading to the induction of EndMT and proliferation; the regulatory roles of these two transcription factors seem to depend on the tissue type [82]. Interestingly, there appear to be differences between in vitro and ex vivo CEC responses to FGF-2 stimulation, as the study of Lee et al. has shown [82]—EndMT occurred 14 days after FGF-2-treatment of ex vivo CECs, whereas in in vitro culture, it occurred after 45 days. Moreover, an increased mRNA and protein expression of vimentin (one of the EndMT markers) occurred in response to FGF-2 treatment in human ex vivo CECs, whereas any changes in vimentin mRNA expression were observed in in vitro CECs. The FGF-2 treatment in the absence of endothelial injury was not sufficient to drive proliferation in CEC [82].

Novel culture media supplements involve inhibitors/activators of selected signaling pathways that significantly improve the outcomes of endothelial in vitro cultures. For example, senescence of CECs, associated with the phosphorylation of p53 and p38-MAPK, can be suppressed by the inhibitor SB-203580 [83]. This substance can also enhance CEC proliferation, as shown on a rabbit wound healing model [84]. EndMT can be avoided using a selective inhibitor of the TGF-β receptor, SB-431542 [85]. The activators of the PI3K/Akt, such as IGF‐1 and heregulin beta (member of the EGF family of proteins), combined with the Smad2 activator—activin A (a member of the TGF-β family of proteins)—were shown to increase the proliferation and migration of cultured rabbit and primate CECs without the occurrence of EndMT [86].

Finally, the ROCK inhibitors such as Y-27632, Y-30141, and Y-39983 can inhibit apoptosis and significantly increase CEC adhesion and proliferation by activating PI3K/Akt signaling, leading to corneal wound healing [87]. The effects of ROCK inhibitors appear to depend on the used cell lines and the culture conditions. For example, some studies have refuted the stimulatory effect of one of the most used ROCK inhibitors, Y-27632, on CEC proliferation but have confirmed its positive effect on CE healing [40, 88]. Nevertheless, due to the overall positive effect of the ROCK inhibitors on CE regeneration, some of them, such as ripasudil and netarsudil, have been approved for treating glaucoma and ocular hypertension [24, 89]. A recent study compared the effects of ripasudil on CEC using several ex vivo models. The study showed that a single dose of ripasudil (30 μM) can upregulate levels of mRNA and protein expression, associated with cell cycle progression, adhesion, migration, CE barrier and pump function, and downregulate the expression of classical EndMT markers (vimentin, ZEB1, SNAI1) in treated CECs [90]. Nevertheless, the specific effects of these promising inhibitors on CE need to be investigated in more detail in the future.

Another type of approach to the in vitro expansion of CECs is the use of a cell-conditioned medium (CM), which acts on CE via various mechanisms. For example, adipose stem cell-derived CM can stimulate CEC proliferation and repair, presumably due to the presence of EGF, FGF-2, and NGF in the CM [91]. The amniotic epithelial cell-derived CM was shown to improve the morphology and proliferative capacity of CECs (via Wnt/β-catenin signaling pathway) and could also reduce apoptosis [92]. The bone marrow mesenchymal stem cell-CM can stimulate CEC proliferation, possibly via FGF-2-activated signaling pathways [93] and/or due to the presence of EGF and IGF 2/6 binding proteins in the CM [94]. Nonetheless, the variability of batches of different CMs represents the main disadvantage of their use because some batches may not have any significant effect on the improvement of CEC cultures under specific experimental conditions [26].

The groundbreaking studies from the last decade reporting the reproducible protocols for in vitro culturing of human CECs (derived from cadaveric corneas) are summarized in Table 2.

**Table 2:** Recent innovative protocols for ex vivo expansion of corneal endothelial cells (from cadaveric corneas)

Cell source	Cell isolation and plating	Culture medium and culture time	Main outcomes	Ref
R-G corneas: n = 269; mean donor age: 53 ± 16 y; DTI: 7 ± 3 d	*• Source tissue*: CE + DM from: whole cornea (Ø 11.5 mm), Cen (Ø 8 mm) or Per (3.5 mm); 1 cornea/1 culture *• Culture models*: explants (n = 13) or cell suspension (P&D method); enzymes tested: 0.1%/0.2% Col II, 0.05% Trypsin/EDTA, 0.1% dispase *• Surface:* 6-well culture plates—uncoated, or coated (collagen IV, fibronectin or FNC)	• Two types of culture media tested: *Medium 1:* EGM-2, 2%-10% FBS, EGF, VEGF, FGF, IGF, HC, Gent, Amp-B; *Medium 2:* DMEM, 10% FBS, FGF • P0: 14 days • P1: successful culture, when cuboidal CECs formed confluent layer at P1 • P2-P3 also performed (enzymes: 0.05% Trypsin/EDTA)	• *Successful cultures*: 31.97% (86 of 269); *• Effect of donor age*: successful (47 ± 18 y) vs. unsuccessful (58 ± 14 y) cultures (p < 0.001) *• Source tissue* (*successful cultures*): whole cornea (47%, 8/17), Per (28%, 64/230), Cen (64%, 14/22), variable sample sizes *• General selection criteria for successful CEC culture*: young donors (≤ 30 y), cell suspension model (0.2% Col II digestion), mitogen-rich medium (e.g., EGM-2), coating substrate (FNC) • CECs from both Cen and Per regions reached confluence and could be passaged (up to P3)	[17]
R-G corneas: n = 33 pairs (mean donor age: 20 y; DTI: 5–14 d) + n = 4 single corneas (age: 60–66 y; DTI: 4–8 d); ECD ≥ 2000;	*• Source tissue*: paired corneoscleral tissues; 1 cornea/1 culture condition; *• Culture model*: P&D method—peeling of CE + DM from whole cornea + enzymatic digestion: 2 mg/ml Col A and 1X TrypLE *• Surface*: FNC	• Overnight pre-stabilization of isolated CECs in *SM* (Endo-SFM + 5% FBS) *• Dual-media: PM* (M199 + F12, 5% FBS, 20 mg/ml Asc, 1X ITS, 10 ng/ml bFGF); effect of Y-27632 (3**–**1000 µM in *SM*) also analyzed • P0: *PM* (∼14 d) + *SM* (7 d) • P2 or P3 used for experiments • subculture (enzyme: TrypLE)	• Dual-media culture led to confluent monolayers with polygonal/hexagonal CECs that expressed Na^+^/K^+^-ATPase, ZO-1, GPC-4, CD-200, detected by IHC • Y-27632 in culture medium improved adherence, cell morphology, and overall cell yield compared to donor-matched control cultures; optimal concentration of Y-27632 was 10 µM • Y-27632 enhanced cell proliferation in cultures derived from young, but not from old (≥ 60 y) donors	[128]
R-G corneas: n = 24 pairs; mean donor age: 64 ± 14 y; DPT: 17 ± 6 d; DTI: 32 ± 7 d; ECD < 2200	*• Source tissue:* paired corneas/1 culture *• Culture model*: P&D method—peeling of CE + DM from whole cornea + enzymatic digestion: 2 mg/ml Col I and 1X TrypLE *• Surface:* Lab-Tek II, coated with FNC • *Plating*: 100µL of the cell suspension per well	*• Dual-media: PM* (M199 + F12, 5% FBS, 1% Asc, 0.5% ITS, 25 µg/mL rh-bFGF, 10 µM Y-27632, antibiotics); *SM* (Endo-SFM, 5% FBS, antibiotics) • P0:∼15 d; no passages; *• 4-plating conditions:* 2 conditions: cells covered with sodium hyaluronate after plating (forced attachment) + 2 conditions without forced attachment	*• Effect of forced attachment:* CECs grew faster in *PM* in the first week of culture (average ECD 2500 ± 94 cells/mm^2^ at day 15), compared to CECs grown without forced attachment; CECs cultured with forced attachment were vital (low level of apoptosis), highly metabolically active, and had a higher number of focal adhesions (vinculin) compared to untreated controls *• Conclusion:* CECs derived from older donor corneas can be effectively expanded ex vivo, and the outcome of such culture can be improved by forced attachment, using non-toxic viscoelastic solution	[45]
R-G corneas: n = 35 pairs; DTI: 10 d (median); ECD ≤ 2200	*• Source tissue:* paired corneas/1 culture; *• Culture model*: P&D method—peeling of CE + DM from whole cornea + enzymatic digestion: Col I, or Liberase, or TrypLE *• Surface:* coatings tested: FNC, collagen IV, rLaminin-511/521; *• Animal model for Tx:* rabbits with induced bullous keratopathy	• Pre-stabilization (≤ 48 h) of isolated CECs in *SM* (Endo-SFM + 5% FBS) *• Dual-media: PM* (M199 + F12, 5% FBS/EquaFetal/hu-serum, 20 μg/ml Asc, 1 × ITS, 10 ng/ml bFGF); *SM* (Endo-SFM, 5% serum) • P0: *SM* (2d) + *PM* (∼14 d) + *SM* (2 d) • P2-P3 used for experiments (IHC etc.) • P1-P2 used for preparation of T-E graft = CECs seeded onto decellularized DM/stroma lenticule at ECD 3,000 and kept in *SM* for 5**–**7 d prior Tx)	*• Effect of enzymes*: Liberase performance was comparable to Col I *• Surface coatings:* attachment profiles of collagen IV and rLaminin-511/521 were similar to FNC, but laminins (mainly rLaminin-511) were better substrates than collagen IV and FNC *• Effect of serum:* FBS and EquaFetal—similar performance; hu-serum led to inconsistent cell growth, worsened morphology and fibroblastic changes of CECs during culture *• IHC:* Human CECs (up to 3rd passage) formed CE monolayer, and expressed Na^+^/K^+^-ATPase, ZO-1, CD166, PRDX-6 + had normal karyotype *• Tx results:* functional mosaic of CE and stromal hydration re-established at day 28 post-Tx into the rabbits' eyes	[30]
C-G corneas: n = 36, donor age: 10–70 y (74% donors ≤ 40 y old); DPT: ≤ 24 h; DTI: ≤ 14 d; ECD ≥ 2500 (89% corneas)	*• Source tissue*: 1 cornea/1 culture *• Culture model*: P&D method—peeling of CE + DM from whole cornea + overnight pre-incubation of lamella in *PM* + enzymatic digestion: 0.02% EDTA *• Surface* 12-well culture plate (1–3 wells) coated with FNC	• *Dual-media*: *PM* (Opti-MEM I, 8% FBS, 5 ng/mL rh-EGF, 20 ng/mL rh-NGF, 100 µg/mL BPE, 0.5 mM L-Asc 2-P, 200 mg/L CaCl_2_, 0.08% CHS, 50 µg/mL Gent, 1X AA); other tested supplements: L-Asc, Y-27632, SB-154352, rh-R-spondin-1 *• SM: SM1* (Endo-SFM, 4% FBS, 50 µg/mL Gent, 1X AA); *SM2* (Opti-MEM I, 4% FBS, 50 µg/mL Gent, 1X AA) • P0: *PM* (∼2**–**4 weeks) + *SM* (7 d) • P2**–**P4 used for experiments	*• Effect of ascorbate:* higher cell counts in medium with L-Asc 2-P than in the media with L-Asc; *• Y-27632:* no effect on CECs proliferation; higher doses of the inhibitor led to EndMT *• SB-154352:* no effect on proliferation or survival of CECs *• R-spondin-1:* no significant effect on proliferation of CECs *• SM: SM1* better than *SM2*; *SM1* supported canonical cell morphology (expression of CD56 marker) and barrier function of CE; CECs maintained in *PM* for a prolonged period: low CD56 expression and initiation of EndMT	[40]
C-G corneas; donor age: 7–29 y; DTI: ≤ 14 d; ECD ≥ 2500; Note: first in-human clinical trial	*• Source tissue*: 1 cornea/1 culture *• Culture model*: P&D method—peeling of CE + DM from whole cornea + enzymatic digestion: 1 mg/mL Col A *• Surface*: 6-well culture plate (2 wells coated with collagen type I) *• Patients (Tx)*: 11 patients (age: 20**–**90 y) with bullous keratopathy	*• Proliferation medium only: PM* (Opti-MEM I, 8% FBS, 200 mg/L CaCl_2_, 0.08% CHS, 50 mg/mL Gent, 5 ng/mL EGF, 20 µg/mL Asc, 10 µM Y-27632; 10 µM SB-203580, 1 µM SB-431542) • P0: *PM* (∼4 weeks); • P2-P3 used for Tx	• Cell lots for clinical application were examined to verify that they met criteria for surgical use and 1 × 10^6^ (Patients 2**–**11) or 5 × 10^5^ (Patient 1) CECs resuspended in 300 μl of modified Opti-MEM I medium with Y-27632 were injected into the anterior chamber of bullous keratopathy patients; patients were kept in a prone position for 3 h to enhance the adhesion of the injected CECs • 24 weeks after cell injection, ECD increased in all (11/11) patients and visual acuity improved in most patients (9/11) • Prospective observational study confirmed that at 5 years after surgery, the CE function was restored in 10 of the 11 eyes, whose mean central corneal ECD was 1257 ± 467 cells/mm^2^	[24]
R-G corneas: n = 18 pairs (mean donor age: 19 y) + n = 6 single corneas (donor age: 19–69 y); DTI: 9 d (median); ECD ≤ 2200	*• Source tissue:* whole cornea; *• Culture model*: P&D method—peeling of CE + DM from whole cornea + enzymatic digestion: Col I, and TrypLE *• Surface:* collagen IV, seeding density: 1 × 10^4^ cells/cm^2^ *• Animal model (Tx):* rabbits (n = 20) with bullous keratopathy *• Tissue-engineered (T-E) group: Group A* (n = 3, received T-E graft), *Group B* (n = 3, no DM + no T-E graft)*, Group C* (n = 3, T-E graft = DM/stroma lenticule with no cultured CECs) *• Cell-injection (C-I) group: Group 1* (n = 5, no CE, intact DM, C-I graft), *Group 2* (n = 3, no CE, no DM, C-I graft), *Group 3* (n = 3, no CE, intact DM, treatment: only Y-27632)	• Overnight pre-stabilization of isolated CECs in *SM* (Endo-SFM + 5% FBS) *• Dual media: PM* (M199 + F12, 5% serum, 20 μg/ml Asc, 1 × ITS, 10 ng/ml rh-FGF); *SM* (Endo-SFM, 5% serum) • P0: *SM* (1d) + *PM* (∼2–4 weeks) + *SM* (≤ 48 h) • P2-P3 used for experiments; *• T-E graft:* P1-P2 CECs seeded onto decellularized DM/stroma lenticule at ECD 3,000 (∼8.5 × 10^4^ cells) and kept in *SM* for 5**–**7 d prior Tx *• C-I graft:* approx. 6.0 × 10^5^ CECs (P2) re-suspended in 150 μl of *SM* + Y-27632	*• IHC, flow-cytometry:* CECs (P2-P3) expressed Na^+^/K^+^-ATPase, ZO-1, CD166, PRDX-6 *• Corneal transparency (3 weeks post-Tx)*: *T-E group—Group A*: Cen region of cornea, corresponding to the graft remained clear, but Per region (= no DM, no cells), remained hazy throughout the study period; *Group B*–*C:* both remained hazy; *C-I group*: signs of intraocular inflammation after Tx, which resolved one week after Tx; unlike *Group 2* and *3* corneas, *Group 1* corneas remained clear throughout the follow-up period (21 d) *• Central corneal thickness (at week 3)*: *T-E group* had thinner corneas than *C-I group* (NS difference) *• Mean ECD post-Tx (at week 3): T-E group*: ECD 1248 ± 64; *C-I group*: 1409 ± 128 *• Characterization of excised corneas:* only *Group A (T-E group)* and *Group 1 (C-I group)* CE were reactive to anti-human specific nuclei antibody attributing corneal recovery to the functional human CECs • Both *T-E* and *C-I grafting* can be used for cell-based therapy of diseased CE, but further evaluation of long-term safety and efficacy of the two methods is necessary	[21]
R-G corneas: n = 19; mean age: 72 ± 5 y; DTP: 10 ± 5 h; ECD < 2200	• *Source tissue*: 1 donor/1 culture *• Culture model*: P&D method—peeling of CE + DM from Cen (Ø 8.25 mm) and Per (2.75 mm) regions of CE + enzymatic digestion: 2 mg/mL Col I, 1X TrypLE *• Surface:* Lab-Tek II (FNC); 100 ul cells/1 well	*• Proliferation medium only: PM* (M199 + F12, 5% FBS, 1% Asc, 0.5% ITS, 10 ng/mL rh-bFGF, 10 μM Y-27632, 1% PenStrep) • P0: 9 days; no passages	*• Cen vs. Per:* CECs from both zones reached confluence (18/19 cultures); higher ECD (p < 0.05) in the Per; positive IHC signal for PRDX-6, ZO-1, Ki-67 in both regions; NS difference in proliferation rate, cell area, and hexagonality between regions • One R-G cornea allowed preparation of two full grafts (from Cen and Per) usable for Tx	[39]
R-G corneas: n = 22 (n = 16 pairs + n = 6 single corneas); mean donor age: 53 y; DTI: median 12 d;	*• Method 1*: peeling of CE + DM, pre-incubation in *SM* or *PM* (≥ 48 h), TrypLE digestion, non-propagated CECs collected for Tx (cell-injection) *• Method 2*: peeling of CE + DM, digestion with Col; ex vivo propagation of CECs prior cell injection; pooled cells from 1 paired donor corneas/1 culture *• Animal model for Tx:* rabbits (n = 20) with bullous keratopathy	*• Dual-media (Method 2): PM* (M199 + F12, 5% FBS, 20 μg/ml Asc, 1 × ITS,10 ng/ml bFGF); *SM* (End-SFM, 5% FBS, antibiotics) • *Tx*: 6 × 10^5^ cells CECs (P2) collected and resuspended in 150 µL of *SM* with Y-27637 for injection into the rabbit eyes	*• 48-h pre-incubation of peeled CE* + *DM lamellas in SM vs PM*: unlike *PM*, the *SM* improved cell morphology and cellular yields after cell isolation *• Tx of CECs prepared by Method 1* and *Method 2:* both methods were comparable**–**both treated groups of rabbits formed CE monolayer and maintained corneal clarity after Tx throughout the study period; corneas that did not receive any cells remained significantly (p < 0.05) thicker compared to corneas treated with cells (*Method 1* and *2*) • Authors showed that a direct collection of a good quality CECs from donor R-G corneas is possible and allows utilization of R-G corneas for Tx purposes	[20]
R-G cadaveric corneas: n = 28; mean donor age: 18 y; DPT: ˂ 24 h; DTI: ˂ 15 d; ECD ≥ 2300	*• Culture model:* P&D method—peeling of CE + DM, followed by two variants of isolation: *Method 1* (trypsin + laminin coated surface), or *Method 2* (Col A + Collagen IV coated surface)	• Two types (conditions) of cell culture: *Condition 1 (Single medium): PM* (M199 + F12 medium, FBS, rh-insulin, Asc, PenStrep, rh-bFGF) or C*ondition 2 (Dual media): SM* (Endo-SFM, FBS, PenStrep, Amp-B) and *PM* (M199 + F12, FBS, L-Asc-2-P, PenStrep, ITS, Amp-B, rh-bFGF) • P0 until confluence • P1-4 also used for experiments	*• Condition 1 vs. Condition 2:* CECs (P0) maintained in *SM* (*Condition 2*) had a robust expression of the CEC-specific genes in P0, i.e., 85.6% (87/97) of the selected CEC-specific genes were expressed, while CECs (P0) cultured in *Condition 1* expressed only 78.4% (76/97) genes • Continuous passaging induced replicative cell senescence and loss of CECs identity, e.g., by P4, only 75.3% (73/97) CEC-specific genes were expressed in *Condition 2*-cultured CECs • SLC4A11 and CD44 may represent the optimal markers of high-quality cultured (by *Method 2* + *Condition 2*) CECs	[18]

### Endothelial-to-mesenchymal transformation

**Fig. 4:**
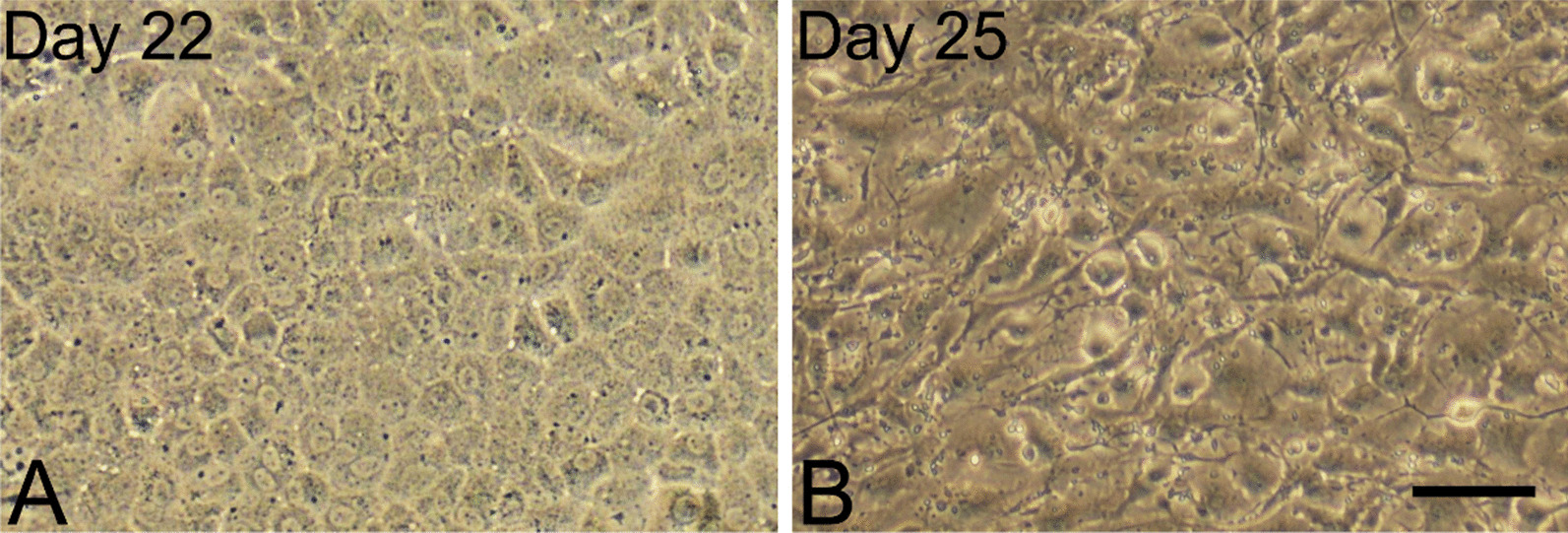
Healthy corneal endothelial cells (**A**) and corneal endothelial cells undergoing endothelial-to-mesenchymal transformation (**B**). In vitro cell culture lasting 30 days; phase-contrast microscopy. Bar: 100 µm. Source: Authors’ (IS, KJ) archive

As mentioned previously, EndMT is a major problem associated with the preparation of many CEC cultures [81, 82]. During EndMT, CECs lose their apical-basal polarity and acquire a migratory (myo)fibroblastic phenotype (Fig. 4), which is associated with the massive reorganization of cytoskeletal components, changes in gene expression, and CE function loss [81]. EndMT accompanies pathological wound healing of the cornea. It can manifest clinically as a retrocorneal fibrous membrane between the CE and DM after graft failure following endothelial keratoplasty [95]. Endothelial diseases, such as Fuchs endothelial corneal dystrophy [96] or congenital hereditary endothelial dystrophy [97], involve EndMT as well.

At the cellular level, EndMT leads to a massive reorganization of the actin cytoskeleton, with downregulation of the junctional protein epithelial (E)-cadherin and the upregulation of neural (N)-cadherin, which translocates from the cell membrane to the cytoplasm, a phenomenon termed the “cadherin switch”; this process alters adhesion and enables the migration of transformed CECs [98]. During EndMT, the β-catenin is released from its association with E-cadherin and is translocated to the nucleus, where it can initiate the expression of characteristic EndMT markers (α1/α2 type I collagen chains, α-smooth muscle actin, vimentin, etc.) [81]. It appears that β-catenin must be overactivated by external signals (e.g., TGF-β) and must reach a particular threshold for the onset of EndMT [81, 99]. During EndMT, actin-rich membrane protrusions (lamellipodia, filopodia) and actin stress fibers are formed (via ROCK signaling) [81].

Triggering factors leading to EndMT include the integration of multiple signaling pathways, such as 1) canonical Wnt/β-catenin signaling, 2) TGF-β/Smad and TGF-β/non-Smad signaling, 3) the FGF-2/SNAI1 pathway, or 4) Notch signaling [81, 100]. The various EndMT inhibition strategies developed in recent years are based on the inhibition of these signaling pathways. For example, EndMT activation via the Wnt/β-catenin pathway could be inhibited by treating CECs with p120 siRNA [58]. The inhibitors SB-431542 or LY-2109761 were successfully used to avoid EndMT induced via TGF-β signaling [85, 101]. Recently, Li et al. [102] showed that the topical application of nicotinamide to mechanically damaged rabbit CE not only promotes CEC proliferation but also inhibits TGF-β1-induced EndMT. Another substance—marimastat (a matrix metalloproteinase inhibitor)—was found to suppress EndMT in CECs cultured with FGF-2 [103]. EndMT can also be suppressed by synthetic peptides corresponding to sequences in the ECD4 region of N-cadherin [104]. Nevertheless, a topography of culture substrate seems to modulate EndMT in in vitro propagated CECs as well [61].

Besides EndMT, CECs can also acquire an abnormal epithelial-like morphology. This process has been referred to as endothelial-to-epithelial transition (EndET) and is associated with posterior polymorphous corneal dystrophy (PPCD) disease [105]. In PPCD corneas, epithelial-like features are present, such as stratification of the normal CE monolayer and the expression of epithelial keratins (K7, K19) [106]. Some endothelial-specific genes can be abnormally up- or downregulated [105]. A master regulator of EndMT, the transcription factor ZEB1, apparently also regulates EndET [105]. The targeted inhibition of ZEB1 using interfering RNAs and/or other small chemicals may reverse EndMT/EndET, but so far, no direct therapeutics have been developed because it is difficult to suppress the activities of this transcription factor [107].

## Quality assessment of endothelial grafts

Cell-based therapy requires the targeted screening of the phenotype and functionality of CECs in the tissue-engineered grafts to ensure that the cells are of adequate quality for Tx. Molecular phenotypic markers are mainly used for this purpose, as well as tests verifying the barrier and pump function of the cultured endothelium; the prepared CE graft’s function can be evaluated by a trans-endothelial electrical resistance assay [108].

Several whole-genome expression studies of ex vivo human CECs published to date [28, 109] have identified potential CEC-specific markers, identifying healthy CECs, as shown in Table 3. Only a limited number of transcriptomic analyses have been reported for in vitro primary cultured CECs [15, 18, 109]. This issue could be problematic because it has been noted that there may be differences between in vivo, ex vivo, and in vitro CECs [15, 82].

**Table 3:** Recently suggested phenotypic markers of healthy and transformed CECs that can identify the endothelial phenotype

Cell marker (gene)	Molecular family	Function	Ref
**Healthy CEC phenotype**			
Cadherin-2/N-cadherin (*CDH2*)	Transmembrane protein	Regulates contact inhibition, proliferation, and EndMT. Proteomic analysis confirmed its exclusive expression in ex vivo CECs	[18, 33]
CD56/neural cell adhesion molecule 1 (*NCAM1*)	Glycoprotein	Cell adhesion, cell interactions, migration, embryogenesis; a functional marker of the ability of CECs to form tight junctions. Proteomic analysis found *NCAM1* expression also in ex vivo corneal stromal keratocytes	[18, 31, 108]
CD98/large neutral amino acid transporter (*SLC3A2* + *SLC7A5*)	Heterodimeric transmembrane glycoprotein	Sodium-independent amino acid antiport, transportation of non-amino acid substrates across the cell membrane. Proteomic analysis found *SLC3A2* gene expression also in ex vivo corneal stromal keratocytes and epithelial cells	[18, 129]
CD166 (*ALCAM*)	Immunoglobulin receptor	T-cell activation and proliferation maintain tissue architecture, mediate homotypic interactions with other ALCAMs. Proteomic study confirmed its specificity to ex vivo CECs	[18, 130]
CD340/receptor tyrosine protein kinase erbB-2 (*ERBB2*)	Cell membrane tyrosine kinase	Binds to other ligand-bound EGF receptors, stimulating cytoplasmic kinase activation and transphosphorylation. Proteomic analysis found *ERBB2* gene expression also in ex vivo corneal stromal keratocytes and epithelial cells	[18, 129]
Sodium bicarbonate transporter-like protein 11 (*SLC4A11*)	Transmembrane protein carrier	Cotransporter that is highly expressed in in vivo and in vitro CE and is critical for CEC function. Its expression in CECs decreases with the increasing in vitro passages and also at high mitogenic conditions. Proteomic study found its expression mainly in ex vivo CECs, but small expression was also in ex vivo keratocytes	[18, 109]
Transmembrane Protein 178A (*TMEM178A*)	Transmembrane protein	A negative regulator of osteoclast differentiation in basal and inflammatory conditions. A specific cell surface marker expressed in early passages of human CECs (donors: ˂ 40 years old)	[15, 18]
**Transformed (fibroblastic) CEC phenotype**			
CD24 antigen (*CD24*)	Sialoglycoprotein	Cell adhesion molecule that may have a pivotal role in the differentiation of different cell types. CD24^+^ subpopulations of cultured human CECs contain chromosomal aberrations (trisomy)	[131]
CD44 antigen (*CD44*)	Glycoprotein	Receptor binding ECM components important for cell–cell interactions, cell migration, and maintenance of stem cell features. Expressed in ex vivo corneal epithelial cells and keratocytes; its expression in in vitro cultured CECs increases with the increasing passages. CD44^+^ subpopulations of cultured human CECs contain chromosomal aberrations (trisomy)	[18, 131]
CD105 antigen/endoglin (*ENG)*	Glycoprotein	Regulates angiogenesis; TGF-β coreceptor involved in the TGF-β/BMP signaling cascade. According to the proteomic study, it is also present in ex vivo CECs	[18, 131]
CD109 antigen (*CD109*)	Glycoprotein	Binds and negatively regulates TGF-β signaling. Increased expression in cultured human CECs with modified (non-canonical) morphology and EndMT cells	[108]
CD133 antigen/prominin 1 (*PROM1*)	Transmembrane glycoprotein	Cell differentiation, proliferation, and apoptosis; bind cholesterol, cadherin, and actinin. The flow cytometry analysis of surface markers identified CD166^+^/CD133^−^/CD105^−^/CD44^−^/CD26^−^/CD24^−^ subpopulations of cultured human CECs as the most suitable cells for Tx	[26]

**Fig. 5:**
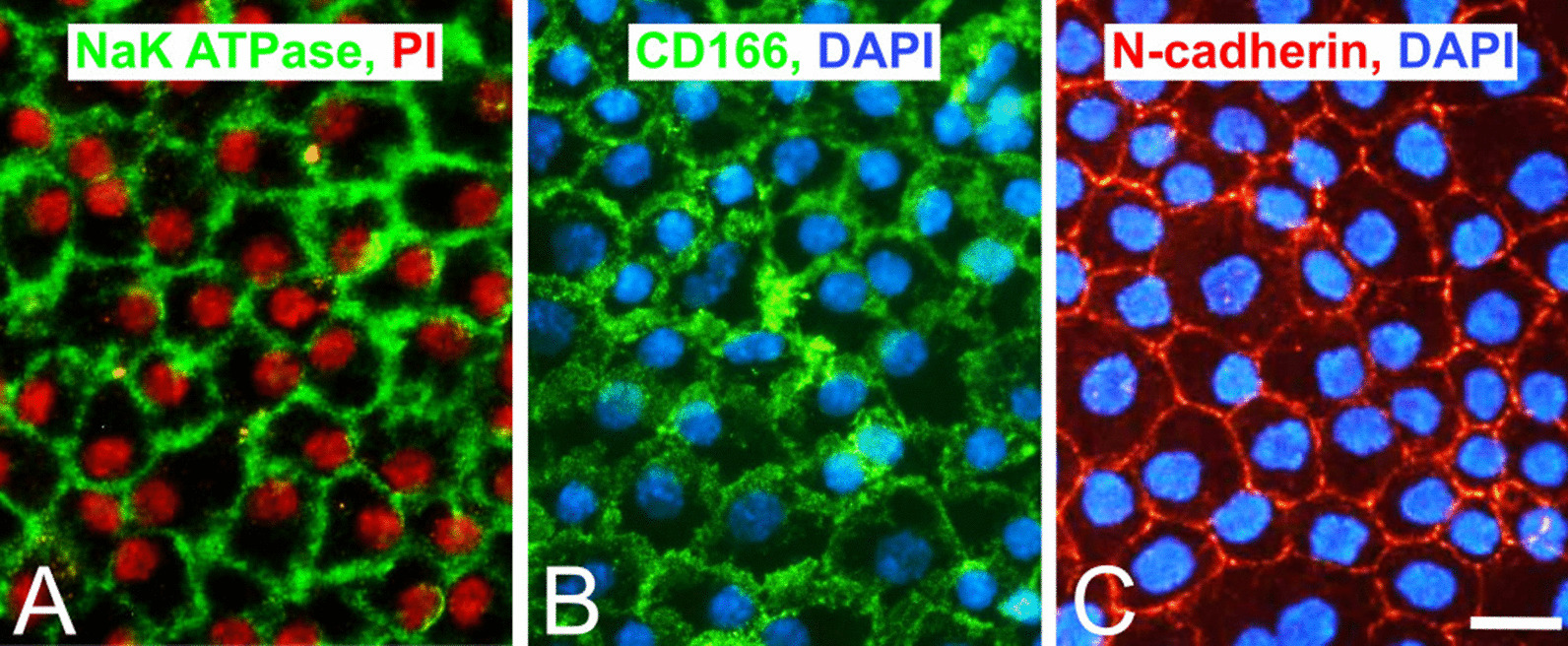
Corneal endothelial surface protein immunofluorescence staining. A classical endothelial marker, the Na^+^/K^+^-ATPase pump (green) (**A**), and “novel” markers CD166 (green) (**B**) and N-cadherin (red) (**C**) in healthy intact human corneal endothelium; impression cytology. Scale bar: 20 µm. Source: Authors’ (IS, KJ) archive

To date, no unique, robust marker for CEC identification has been identified. The standard phenotype markers (i.e., Na^+^/K^+^-ATPase and zonula occludes (ZO)-1) are not specific to CECs because they can be found in other corneal cells, such as the corneal epithelium. Recent studies have identified potential markers of healthy CEC populations (SLC4A11, N-cadherin), as shown in Fig. 5, and populations of unhealthy/transformed CECs (CD44) [18, 26], as shown in Table 3.

Alternatively, the quality of cultured CECs was also assessed by screening of the microRNA expression profiles in cultured CECs, with the miR-34a identified as the only miRNA capable of discriminating healthy (CD44 negative) from transformed (CD44-positive) CEC populations [27]. However, a prerequisite for this approach is to exclude the origin of these miRNAs from sources other than the CECs themselves.

## Storage of endothelial grafts

The correct storage of T-E grafts (cell suspension or T-E lamella) is a prerequisite for maintenance of the graft quality. Without long-term storage of grafts (e.g., via the cryopreservation method), it would be necessary to deliver the cultured CECs for Tx from the tissue establishment/bank to the surgeon in a short time. Thus, several protocols for storing and shipping cultured CECs have been introduced in recent years.

Bartakova et al. [40] tested the storage of cultured CECs at 37 °C and their overnight shipment in the form of a cell suspension in a temperature-controlled container at 2–8 °C. Then, the cells were kept at room temperature (23 °C) for 72–96 h, which led to a CEC viability of approximately 81%. Resuspended in culture medium, the cells expressed the function-associated endothelial marker CD56.

In another study, Wahling and colleagues [68] examined adherent and suspension storage models for CECs and showed that storing the cells under HT for 2 days in Endo-SFM, followed by 48-h post-storage cell stabilization at 37 °C, led to functional CECs with a well-preserved morphology. Adherent CECs could be stored longer (4 days at 23 °C) than cells in suspension, but transporting adherent cells can be logistically challenging [68].

Cryopreservation can be successfully used for the long-term storage of cultured CECs [30]. However, during cell freezing, even at a controlled cooling rate, cryo-induced cell damage occurs, the extent of which depends on the preservation protocol and the cells’ initial quality [110]. Thus, various cryoprotective storage media have been developed in recent years. The most common cryoprotectant—dimethyl sulfoxide (DMSO)—has been used in several studies. For example, studies on porcine CECs have shown that viable cells can be cryopreserved in a medium containing DMSO and FBS or hydroxyethyl starch (HES), a non-penetrating cryoprotectant that draws water from cells, preventing intracellular ice nucleation leading to cell damage [111]. Eskadari et al. obtained a high post-thaw CEC viability (> 90%) and preserved metabolic activity (12 h post-thaw) when they cryopreserved an in vitro prepared monolayer of porcine CECs on fibronectin-coated vinyl plastic coverslips in DMSO, with HES and chondroitin sulfate, at a controlled cooling rate 0.2–1 °C/min [111]. Xia et al. [31] cryopreserved cultured human CECs (after passage 3) in DMSO (at − 80 °C for 5 days, then stored in liquid nitrogen for 3 more days), following previously developed protocol [40], and loaded the thawed cells with magnetic nanoparticles. After being transplanted into rabbit eyes, the cells formed a functional CE and reduced the corneal edema [31].

In Peh and colleagues' study [30], cultured human CECs (from the third passage) were cryopreserved in 10% DMSO in Endo-SFM with 5% FBS and were compared to CECs that were cryo-preserved in two other DMSO-free cryo-preservation ready-to-use solutions (medium 1 and 2). The cells were cryopreserved at − 80 °C overnight and then stored in liquid nitrogen for at least one week. The post-thawing viabilities of the CECs were as follows: 83.3% ± 3.1% (medium 1), 77.7% ± 10.9% (DMSO), and 72.2% ± 6.4% (Medium 2). After thawing, primary CECs cryo-preserved in all three preservation solutions could be propagated in vitro and formed a CE monolayer of compact polygonal/hexagonal CECs. The long-term survival of CECs, preserved in the best cryopreservation medium (Medium 1) for 24 months, was between 66.3% and 71.8% after thawing [30].

Recently, Okumura et al. [112] provided an alternative to DMSO solution because they obtained good post-thaw viability (89%) of cultured human CECs stored in a commercial serum-free freezing medium for 24 h at − 80 °C, followed by 13 days at − 196 °C. The promising results of these studies suggest that the cryopreservation of T-E grafts will soon become a standard procedure that will contribute to the increased availability of endothelial grafts.

## Transplantation of bioengineered corneal endothelium

Endothelial cell-based therapies include two options, either lamellar keratoplasty (transplantation of T–E CECs expanded on a suitable support) or cell-injection therapy (injection of a suspension of ex vivo cultured CECs into the recipient's eye), both methods being able to restore CE function [21] but each with pros and cons.

The model of transplantation of T-E lamellae, composed of CECs cultured on a support, is based on long-term experience with a standard lamellar keratoplasty performed worldwide. The advantage of the T-E graft is that the patient is transplanted with cells adhered to the substrate that have formed a CE monolayer with the desired characteristics. The disadvantage is that the substrate must meet specific characteristics, such as structural regularity and reproducibility, biocompatibility, and biodegradability (if it is intended only as a cell carrier), or similarity to DM (if it is to be maintained and possibly replace the DM following Tx). A suitable substrate should also support the function of CECs [64].

On the other hand, direct injection of cells into the anterior cornea avoids the difficulties associated with the preparation and handling of the fragile T-E CE layer. The possibility of applying the cell suspension directly to the recipient's eye to restore CE has been successfully tested in animal [72, 113] and ex vivo Tx [114] models. After cell injection, no adverse effects, such as abnormal accumulation of injected cells and clogging of the drainage channel, increased intraocular pressure, or rejection of cells were detected [24, 115]. After Tx, cell attachment was improved by the recipient being in a prone position [72], or magnetic attraction of CECs with endocytosed magnetic nanoparticles [116], which did not appear to impair CEC function and vision recovery [31, 113].

The results of the first human clinical trial in Japan (UMIN000012534), involving cell-based injection therapy, were groundbreaking [24], as given in Table 2. In that trial, the Kinoshita group harvested CECs from young donors (aged 14 to 29 years) and cultured them in a mixture of Opti-MEM I, FBS, EGF, Y-27632 (ROCK inhibitor), SB-203580 (p38-MAPK inhibitor), and SB-431542 (TGF-β inhibitor). Then, the cultured CECs were harvested in the form of a cell suspension and injected into the eyes of 11 patients with bullous keratopathy. Twenty-four weeks after Tx, 100% of treated patients experienced improved visual acuity without any severe adverse reaction [24]. A prospective observational study confirmed that at 5 years after surgery, CE function was restored in 10 of the 11 eyes, the mean central corneal ECD of which was 1257 ± 467 cells/mm^2^. This follow-up study confirmed the safety and efficacy of the developed cell-injection therapy method for treating bullous keratopathy patients [25].

In a different (case) study, human CECs, expanded in vitro by a sphere forming assay, were successfully used for treating three patients with bullous keratopathy [117]. CECs were grown for 26 days on a thermoreversible gelation polymer hydrogel. Prior to cell injection, the nanocomposite hydrogel sheet (D25-NC), composed of organic polymers and a water-swellable inorganic clay, was implanted into the anterior cornea and served as a supporting material for the attachment of cells injected in the gap between the posterior cornea and hydrogel sheet. D25-NC was removed three days after Tx. The bullae in the cornea disappeared in all patients, and visual acuity improved in two patients at 18 months’ follow-up [117].

These initial studies indicate that cell therapies may be a promising alternative to standard lamellar keratoplasty, but the existence of intact DM appears to be an important prerequisite for successful CE layer formation after Tx because injected CECs are unable to form CE and restore vision on bare stroma [21]. However, several major obstacles remain to be addressed before standardization of the cell-based therapy methods in clinical practice.

## Conclusion and future perspectives

Advanced tissue-engineering methods, including novel culture media and biocompatible and biodegradable substrates, allow the utilization of research-grade corneas, even from older donors, for clinical applications. The introduction of a dual-media culture method can be considered to be, so far, the most successful method for reproducible and more massive endothelial cultivation.

Most of the culture protocols presented in the last decade are not xeno-free. Therefore, they do not satisfy clinical-grade graft requirements. Moreover, mass production of corneal endothelial cells, derived from cadaveric corneas, is still not possible due to persistent issues associated with culturing CECs, such as the natural low proliferation capacity of endothelial cells, phenotypic heterogeneity of ex vivo expanded cells, cellular senescence (particularly in cultures derived from older donors), or frequent endothelial-to-mesenchymal transition occurring during culture. Recently introduced culture media supplements, such as selective ROCK or TGF-β inhibitors, represent an option for managing the majority of problems associated with endothelial cell cultures because their suitability has been demonstrated in the first-in-human clinical trial initiated in Japan. However, because components of culture media fall under national legislations, the development of a globally standardized protocol for ex vivo endothelial cell propagation will be difficult.

Cell grafts for clinical use must contain only populations of well-identified endothelial cells capable of forming a functional endothelial monolayer. Because many previously published studies exploring the production of such CE grafts have used established, nonspecific markers, such as Na^+^/K^+^-ATPase or ZO-1, for identifying the corneal endothelial phenotype, future verification of the results of these studies is necessary, implementing novel, more specific phenotypic markers.

An option to prepare corneal endothelial grafts from non-eye native cell populations, such as induced pluripotent stem cells, remains possible because culture protocols for the expansion of these cells are still being developed. However, several issues associated with their use must be solved. In addition, other treatment options for endothelial dysfunctions exist and are continuously being improved, including novel lamellar keratoplasty techniques, topical drugs, or gene therapy methods.

## Literature search method

The PubMed and Google Scholar databases were searched with the following search terms: human corneal endothelium, marker, morphology, cell culture, regeneration, wound healing/repair, storage, endothelial–mesenchymal transition (or transformation), substrate, sphere-forming assay, transplantation, donor parameters, clinical trial, transport, and combinations thereof. The resulting articles in English (2975), published between the years 2010–2021, were reviewed for their titles and abstracts, focusing on the most reliable and cited publications primarily related to human endothelial cultures, derived from human cadaveric corneas. The references of the included articles (523) that met our criteria were also scanned to identify additional relevant articles. A new literature search was then performed every 1–2 months to include the most recent reports on the human corneal endothelium. The articles published before 2010, which were included in this study, are original studies bringing new discoveries about the endothelium, which were confirmed by later studies.

## Data Availability

Not applicable.

## Abbreviations

AA: Antibiotic/antimycotic solution
Amp-B: Amphotericin-B
Asc: Ascorbic acid
Asc-2P: Ascorbic acid 2-phosphate
BPE: Bovine pituitary extract
CD200: Cell membrane glycoprotein
CE: Corneal endothelium
CE + DM: Lamella(s) with Descemet's membrane and attached corneal endothelium
CECs: Corneal endothelial cells
Cen: Central (region of endothelium)
C-G corneas: Clinical-grade corneas = corneas suitable for transplantation
C-I: Cell injection (= in vitro expanded CECs collected and used for transplantation via cell-injection therapy)
CM: Cell-conditioned medium
Col: Collagenase
DM: Descemet's membrane
DMEM: Dulbecco’s modified Eagle’s medium
DMSO: Dimethyl sulfoxide
DPT: Death to preservation time
DTI: Death to cell isolation (initiation of corneal endothelial cell culture) time
ECD: Endothelial cell density (in cells/mm^2^)
EDTA: Ethylenediaminetetraacetic acid
EGF: Epidermal growth factor
EGM-2: Endothelial growth medium-2
EndET: Endothelial-to-epithelial transition
Endo-SFM: Endothelial serum-free medium
EquaFetal: A serum produced from animal that has been maintained on controlled diets and living conditions
FBS: Fetal bovine serum
FGF-2/bFGF: Basic fibroblast growth factor
FNC: FNC coating mix
M199 + F12: Medium M199 with Ham's F12 supplement (1:1)
Gent: Gentamicin
GPC-4: Glypican-4
H: Hours
HC: Hydrocortisone
HT: Hypothermic conditions
hu-serum: Commercially available human serum
CHS: Chondroitin sulfate
IHC: Immunostaining/immunohistochemistry
IOP: Intraocular pressure
ITS: Insulin–transferrin–selenium
K: Keratin(s)
Lab-Tek II: Lab-Tek II chamber slides (8 wells, 0.7-cm^2^ culture area)
L-Asc 2-P: L- Ascorbate 2-phosphate
MAPK: Mitogen-activated protein kinase
MEM: Minimal essential medium
NS: Not-significant (statistics)
Ø: Diameter
OC: Organ culture
P&D: Peel-and-digest method of cell isolation
p: P-value (statistics)
P0: Primary culture
P1-4: Passage 1–4
Per: Peripheral (region of endothelium)
PKC: Protein kinase C
PM: Proliferation medium
R-G: Research-grade corneas = donor corneas deemed unsuitable for transplantation to humans
rh-bFGF: Recombinant human basic fibroblast growth factor
ROCK: Rho-associated protein kinase
SB-154352: The selective inhibitor of TGF-β signaling pathway
SB-203580: P38 MAP kinase inhibitor
SB-431542: Transforming growth factor beta inhibitor
S-C: Successful culture
SLC4A11: Sodium borate cotransporter 1
SM: Stabilization medium
T-E: Tissue-engineering/tissue-engineered
TGF-β: Transforming growth factor beta
TM: Trabecular meshwork
Tx: Transplantation
TZ: Transition zone
y: Years
Y-27632: The ROCK inhibitor Y-27632
ZO-1: Zonula occludens-1
